# The Book World of Medicine and Science

**Published:** 1902-09-06

**Authors:** 


### THE BOOK WORLD OF MEDICINE AND SCIENCE.

An Introduction to Dermatology. By Norman Walker, M.D., F.R.C.P.E., with 43 full-page plates, and 47 illustrations in the text. Second edition. (Bristol: John Wright & Co. 1902. Price 9s. 6d. net.)

Dr. Norman Walker is to be congratulated on finding himself so soon called upon to provide a new edition of this work. We are inclined to agree with him in attributing the success of the first edition largely to its simplicity, and to the care with which he has kept to his text, and has refrained from giving way to the temptation to enter deeply into controversies regarding rare diseases. Beginning with a description of the structure of the skin, and a few words about the classification of its disorders, the author gives a good description of the general principles of the treatment of skin diseases, furnishing in a very useful chapter much information regarding the drugs and applications most commonly used by dermatologists. He then takes the various disorders *seriatim*. There is much in the descriptions and the groupings of these maladies which is likely to arrest the attention of the student, and pin it down to the essential parts. His description of eczema, for example, will serve well to impress upon him how far short of completeness is our knowledge of that "rubbish heap of cases." He says "Eczema is the term commonly applied to any wet or scaly, inflammation of the skin, of the cause or nature of which the observer is ignorant," and he goes on to show how large a multitude of conditions the term covers, and to point out that increase of knowledge is not to be sought by way of new definitions of "eczema," but rather by attempts to identify some definite cause or some definite sequence of events by which certain groups of cases may be separated from that chaotic conglomeration. Meanwhile, as we still, for lack of more definite knowledge, have to deal with eczema, it has to be described, and this Dr. Walker does well and fully, as indeed he does with all the other diseases with which he deals. Definiteness and clearness of description are strong points with him. He knows exactly what he means—a virtue sadly lacking with too many teachers—and he possesses a happy knack of conveying his thoughts to others. We may not always quite agree with him in certain details, but those are matters of discussion among specialists, not among students or practitioners who need an introduction to dermatology. By them Dr. Walker will be found an admirable teacher. In his endeavours to make things plain he is greatly aided by the illustrations of which this book contains a considerable number. That they are not, all of them, works of art is manifest, some of the coloured ones especially being somewhat crude; but most of them express their meaning, which is something. As an introduction to the diagnosis and treatment of skin diseases, this book is likely to prove a most useful guide.

The American Illustrated Medical Dictionary of the Terms used in Medicine, Surgery, Dentistry, Pharmacy, Chemistry, and the Kindred Branches, with their Pronunciation, Derivation, and Definition. By W. A. Newman Dorland, A.M., M.D. (London and Philadelphia: W. B. Saunders and Co. 1900. Price 19s. net.)

This is a very useful and complete medical dictionary which fully carries out the claims made for it on the title-page. In addition to the definitions of the several words a good deal of information is given in a somewhat tabular form. Thus under the word "degeneration" we find nearly a column of different kinds of degeneration; under "bandages" a long and well-illustrated list of such appliances, many of which, being commonly described under the names of their inventors, would be meaningless unless they were carefully defined as they are here. Who, for example, knows what is meant by a Theden's bandage, or a Hueter's or a Heliodorus's bandage? Of bacilli, again, there are many pages, also illustrated. Besides other illustrations there are 24 coloured plates, some of which are remarkably good. The whole book is admirably printed and bound, and is most comfortable and pleasant to read. Perhaps one might take exception to the pronunciation given to some words of French origin. Who, for example, would recognise "bu-baw'-dah-bla'" as meaning *bubon d'emblée*? But take it altogether the book is wonderfully free from errors, and is sure to be found useful.

The Case for Vaccination. By E. Brown, M.R.C.S., L.R.C.P. (London: Baillière, Tindall, and Cox. 1902. Pp. 44. Price 1s.)

Mr. Brown has written a pamphlet which, though intended primarily for the public, will be of interest and profit to the profession. He states the "case for vaccination" clearly, forcibly, accurately, and yet temperately. He avoids ambiguity and dogmatism alike; and he does not fall into the pitfall so damaging to medical controversialists—that of making assertions which, while probably, if not certainly, true, cannot as yet be proved to be true. Mr. Brown admits that vaccination is, in England, unpopular, and he finds the explanation of this unpopularity in, firstly, the rebellion of the average Englishman against "authority"; in, secondly, pure ignorance; and in, thirdly, the attitude of medical men towards the discussion by laymen of professional matters. Fourthly, there is the selfishness of parents—their objection to the inconvenience which the vaccination of an infant naturally entails; fifthly, the too great claims made by Jenner and his immediate successors; and, sixthly, the alleged dangers of the operation. Mr. Brown, after discussing these points, gives an historical résumé of interest and value, and proceeds, after proving the decline of small-pox as a cause of death, to eliminate those factors, other than vaccination, which have been alleged as causes of this admitted decline. The facts and figures put forward by those who have had experience in small-pox hospitals are next discussed, and finally the alleged dangers of vaccination are dealt with. This portion of the book is exceptionally well done; statistics appeal but little to the "man in the street"—he dismisses them with the remark that "figures prove anything." What he wants to know is, "Will he have a bad arm?" "Doesn't vaccination cause cancer?" "What will happen if it takes inwardly?" and so forth. Mr. Brown explains the "danger of vaccination" clearly and sensibly, minimising nothing, extenuating nothing, and advancing no untenable propositions or claims. He deserves the thanks of his profession for a book which stands out amongst the flood of controversial literature at present swamping our writing-tables, and will be appreciated, we believe, by "pro-vack" and "anti-vack" alike for its candour and perspicacity.

### BOOKS RECEIVED.

Diprose, Bateman and Co.

"The Organisation of the Public Health Department in Dealing with the Epidemic of Small-pox in the Metropolitan Borough of Holborn." By Wilfred Watkins-Pitchford, M.B., F.R.C.S., D.P.H.

Baillière, Tindall and Cox.

